# Assessing emotional health needs of patients with schizophrenia in order to apply more effective therapies to improve their quality of life

**DOI:** 10.1186/1472-6955-14-S1-S3

**Published:** 2015-10-08

**Authors:** Andrea Mallorquí Molina, Mercé Comes Forastero

**Affiliations:** 1 Hospital Clínic de Barcelona, Barcelona, Spain

## Background

People who suffer from schizophrenia experience a worsening of their quality of life if compared to the average population. By getting to know their perceived needs in greater depth, I aim to gather data in order to apply more effective therapies seeking to improve schizophrenic patients' quality of life.

## Methods

Qualitative analysis of the phenomenological perspective through a series of interviews conducted with 9 informants with schizophrenia (DSM-IV) has been completed by using the software Atlas.ti to analyze the qualitative data.

## Results

In this study, eight participants out of a total number of nine, hold the belief that anxiety and depression go hand in hand in the symptomatology that most negatively affects their perceived quality of life. This symptomatology leads to an increase in the consumption of cigarettes and *junk food*, among others, which has a negative impact on their already poor health. The informants express the negative influence that side effects of antipsychotic medications have on their physical health. But they don't consider that their lifestyle can affect the latter. On the other hand, the social stigma and the lack of social resources to establish new social networks are other aspects which negatively affect their quality of life. This last piece of information is relevant in this research given the lack of similar data in the scientific literature.

## Conclusions

Depression and anxiety are the two factors that most negatively affect schizophrenic people's quality of life. A surprising finding is the necessity of establishing social links, whose absence causes great emotional discomfort in their lives. Fighting against social stigma, addressing interventions to the empowerment, establishing new health policies not focused on pharmacological treatment and finally improving the access to the Health Care System are key elements to improve schizophrenic people's quality of life.

